# Use of adductor pollicis muscle thickness in hospitalized or ambulatory patients: a systematic review

**DOI:** 10.1590/1518-8345.2045.2960

**Published:** 2018-06-21

**Authors:** Brunna Gabrielly Ferreira da Silva Soares, Andréa Pereira Vicentini

**Affiliations:** 1 Nutricionist.; 2 PhD, Associate Professor, Faculdade de Ciências da Saúde, Universidade Federal da Grande Dourados, Dourados, MS, Brazil.

**Keywords:** Hospitalization, Nutritional Assessment, Anthropometry, Prognosis, Malnutrition, Ambulatorio en Hospital

## Abstract

**Objective:**

to analyze the use of the Adductor Pollicis Muscle Thickness (APMT) as an anthropometric parameter and prognostic indicator in hospitalized or ambulatory patients.

**Method:**

systematic review carried out the Web of Science, SCOPUS and Lilacs databases.

**Results:**

Twenty-three studies were performed on critical, surgical, oncological, nephropathic and hepatopathic patients, collecting data on bibliographic reference, study site, objectives, number of patients, age group, methodology, main results and conclusion. APMT proved to be a good anthropometric parameter for evaluation of nutritional status in critical patients without edema, and surgical, oncological and nephropathic patients, but presented poor performance for diagnosis of malnutrition in hepatopathic patients. It was a good prognostic indicator for mortality in critical, nephropathic and oncological patients, and also a good predictor of hospitalization in nephropathic patients. There was an association with neurological complications in Hepatic Encephalopathy (HE) in the case of hepatophatic patients, but it was not a predictor of postoperative complications in surgical patients.

**Conclusion:**

APTM was considered a good anthropometric parameter in most clinical conditions, except in patients with liver disease and a good prognostic indicator for mortality in critical, oncological and nephropathic patients, and a predictor of neurological complications in HE. Further prognostic investigation, standardization of cutoff points and evaluation of sensitivity and specificity are required.

## Introduction

Due to the limited use of sophisticated equipment for analysis of body composition in the clinical practice, for the high costs and the experience required in such procedures, anthropometric and laboratory parameters are still used for the nutritional assessment of hospitalized patients^1^. Thus, new assessement methods are needed within the hospital environment, particularly those that are simple, relatively non-invasive, and that have high sensitivity and preserved specificity^2^. 

In this context, a new assessment technique of the muscle compartment called Adductor Pollicis Muscle Thickness (APMT) was introduced in 2004 and it has been used to diagnose muscle loss and consequently, malnutrition^3^ ^,^ ^4^. The incidence of malnutrition lies between 20 and 69% in hospitalized patients, being higher in critical cases and nutritional levels^4^ ^,^ ^5^ that lead to increased muscle fatigue, loss of contraction force and relaxation rate of the adductor pollicis muscle (APM)^1^ ^,^ ^2^. 

The opposition of the thumb is present in several activities of the daily life of humans. Since the APM is also consumed during catabolism and weakened when in disuse, its trophic condition can reflect the routine of an individual^1^. This muscle can indicate changes in the muscle composition of the whole body, including early changes arising from both malnutrition and recovery of nutritional status^2^ ^,^ ^4^. 

The technique to measure the APM was developed and published in 2004 and described as follows: the subject should be seated, with the right hand (RH) on the knee and the elbow flexed at an angle of 90 degrees above the homolateral lower limb. The adipometer indicated by the researchers is the Lange®, which should be used with a continuous pressure of 10g/mm². The evaluator pinches the adductor muscle located at the vertex of the imaginary triangle formed between the extension of the thumb and the index finger. This should be performed in triplicate to obtain a mean APM value. The reference values ​​determined by the author serve to classify the degree of muscle loss in healthy individuals^1^.

The accuracy and reliability of anthropometric measures are influenced by many variables, such as: equipment, technical ability, cooperation of the individual, and variety of reference standards^2^. However, this new technique has many advantages; the APM is a muscle almost devoid of adipose tissue, flat, with a well-defined anatomic point, being the only one that allows the direct measurement of its thickness without need for calculations, and prone to quick, simple, non-invasive, low-cost measurement, easily reproducible by other researchers in both ambulatory and bedridden patients^1^ ^,^ ^2^ ^,^ ^4^ ^,^ ^5^. Thus, the objective of this study is to perform a systematic review on the use of APMT as an anthropometric parameter and prognostic indicator in hospitalized or ambulatory patients, when compared to other methods of assessment of nutritional status. 

## Methods

The following strategy was used for selection of descriptors and formulation of the guiding question of the systematic review. The group of patients named as “P” included hospitalized or ambulatory patients; the intervention “I” consisted in the application of the APMT; the comparison methods “C” were anthropometric and prognostic; and the expected outcome “O” was a good correlation between the evaluated variables. The guiding question of the research is: “Is the APMT a good anthropometric method and prognostic indicator compared to other methods used to evaluate nutritional status in hospitalized or ambulatory patients?” The following Health Sciences Descriptors were used (DeCS) to search scientific articles: “Hospitalização” OR “Avaliação nutricional” OR “Antropometria” OR “Prognóstico” OR “Desnutrição” OR “Ambulatório Hospitalar” combined with the term “Músculo adutor do polegar”, which is not indexed in DeCS, and its English and Spanish translations “Hospitalization”, “Hospitalización”, “Nutritional Assessment”, “Evaluación nutricional”, “Anthropometry”, “Antropometría”, “Prognosis”, “Pronosis” “Malnutrition”, “Desnutrición”, “Servicio Ambulatorio en Hospital”; “Hospital Outpatient Clinics”, “Adductor pollicis muscle” and “Músculo aductor del pulgar”.

The electronic databases used in the search for articles that are part of the systematic review of the scientific literature were the Web of Science (Thomsons Reuters), Scopus platform, and Latin American and Caribbean Literature in Health Sciences (Lilacs). The research and selection of articles were carried out by only one of the authors. Only articles duplicated in the databases were initially excluded. Then, eligibility criteria were adopted for the selection process, which began with the inclusion of studies that had applied the method of assessment of the APMT in hospitalized or ambulatory patients. After this step, titles and abstracts were read to select the texts that included the clinical conditions for the study presented in more than one article (critical, surgical, oncologic, nephropathic and hepathopatic patients), excluding articles with healthy patients, institutionalized elderly, hospitalized patients without pathological specifications, and patients with pathologies presented in only one article.

A thorough reading of the selected studies was performed and the following data were collected: bibliographic reference, study site, objectives, number of evaluated patients, age range, methodology, main results and conclusion. These data were organized according to the clinical conditions abovementioned and later discussed along with the other important considerations reported in the researches. Full texts of articles of which only the abstract and title available were requested from the Federal University of Grande Dourados library, and those that were not found were excluded due to the inaccessibility to the statistical results. The last selection process excluded complete studies for the following reasons: insufficient results and lack of comparison with anthropometric variables and prognostic indicators. 

The anthropometric variables considered for comparison were body mass index (BMI), arm circumference (AC), arm muscle circumference (AMC), total arm area (TAA), arm muscle area (AMA), calf circumference (CC), triceps skinfold (TS), percentage weight loss, hand grip strength (HGS), electrical bioimpedance (EBI) [resistance, reactance, percentage of cell mass and phase angle (FA)] and Dual Energy X-ray Absorptiometry (DEXA). The variables considered with prognostic value for comparison were the Global Subjective Assessment (GSA), Sequential Organ Failure Assessment (SOFA), Glasgow prognostic score, length of hospital stay, days of mechanical ventilation, mortality, complications in the postoperative period (PP), risk of hospitalization, severity of the disease, serum albumin, serum creatinine and hemoglobin. 

## Results

**Figure 1:**
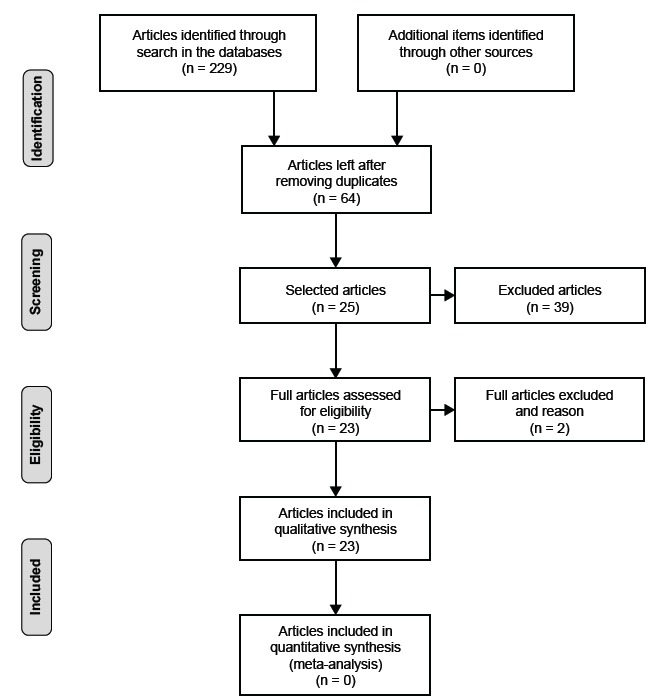
Steps for selection of articles

Two hundred and twenty nine articles were initially searched, of which 165 were repeated and 64 were accessed. After the first selection by the criterion described in the methodology, 36 articles remained. The second selection (reading of the titles, abstracts and choice of articles of interest for research) resulted in 25 articles and the last step resulted in the final selection of 23 articles, 13 from the Web of Science, 6 from the Scopus Platform and 4 from the Lilacs. The studies included in the study were divided into five groups according to the following clinical conditions: critical (N = four), surgical (N = eight), hepathopatic (N = five), oncological (N = three) and nephropathic (N = three) patients. Steps for selection of articles are described in the Figure 1.

The following are the main results found in the studies conducted with critical patients and that evaluated APMT in both hands. 

The first study was conducted in Cuiabá (MS) in 2012 and found that abnormal APMT (values below those ​​found by other researchers) was associated with higher mortality (p = 0.03) and significantly correlated with the GSA outcomes for severe malnutrition (p < 0.001). Only patients without edema had a significant association between APMT measurement and length of hospital stay [APMT of RH without edema (β = -0.32, p = 0.03) and left hand without edema (β = -0.36, p = 0.02)]. It was also observed that patients with abnormal APMT values had ​​stayed in the intensive care unit (ICU) for approximately five days. There was no correlation between APMT and the number of days of mechanical ventilation. The values ​​found were above those found in other studies, being higher in the RH, what the author considered to be due to the presence of edema in the hands^4^.

In 2015, a study carried out in Porto Alegre (RS) verified the accuracy of APMT by evaluating the *Receiver Operating Characteristics* (ROC) with the GSA (area under the curve regarding the RH of 0.82 and 95% confidence interval of 0.73 to 0.91). The study also related measures of less than 6.5 cm to higher nutritional risk in the GSA (moderately and severely malnourished). APMT of both hands was correlated with the BMI (p < 0.05 and r = 0.45) and CC (p < 0.05 r = 0.58); but not with length of hospital stay (p = 0.411) and mortality (p = 0.519). The study population consisted of 73.5% of elderly participants, which explains the low values ​​found^3^.

A study carried out in Asia in 2015 observed a significant difference in APMT measurements between races and genders (p < 0.05). No significant correlation was found with mortality at 28 days, hospital outcome, and ICU length of stay (p > 0.05). However, there was a significant and moderate correlation with AC and BMI (p < 0.05), and APMT values ​​were higher when compared to other Brazilian studies^2^. A study conducted in Iran found a strong correlation between APMT of both hands and all anthropometric variables evaluated, namely, TS, AC, AMC, ATB and AMA (p <0.0001), as well as serum albumin (r = 0.61, p = 0.001). The measures of the dominant hand (DH) presented positive correlation with ICU length of stay (r = -0.4, p < 0.001) and the highest correlation with mortality (odds ratio 3.8, 95% confidence interval, 1.2 to 5.2, p < 0.01). A significant correlation was also found between the dominant APMT and the SOFA score for organ failure (r = -0.86, p < 0.001)^5^.

Other studies evaluated surgical patients, three of which were performed with gastrointestinal (GIT) surgery patients, and the others with patients recovering from major surgeries and elective procedures. 

In a research conducted in Rio de Janeiro (RJ) in 2005, APMT showed a significant correlation with septic complications in the PP in the case of patients with APMT values ​​lower than 6.5 mm (p = 0.007), as well as correlation with mechanical ventilation time (p = 0.000), ICU length of stay (p = 0.026), and significant tendency for hospital admission (p = 0.053). However, there was no significant association with non-septic PP complications (area under the ROC curve of ​​0.562 and p = 0.305) and mortality (area under the ROC curve of ​​0.641 and p = 0.217)^6^.

In the study carried out in Cuiabá (MT) in 2009, APMT was significantly correlated (p < 0.05) with all the anthropometric variables evaluated (BMI, AMC, AC, TS and percentage of weight loss) and had a good correlation with GSA values (p <0.05), considered gold standard. In addition, it presented good sensitivity (72.37% for APMT of the DH and 77.33% for that of NDH) and 100% of specificity in both hands. The cutoff point for malnutrition was determined by the ROC curve, with 13.4 mm for the DH and 13.1 mm for the NDH. In the most recent study in 2011, there was an association between APMT and HGS (DH p < 0.001; NDH p < 0.001) and mortality (p < 0.05). However, there was no significant association with length of hospitalization and complications in the PP (data not shown)^7^ ^,^ ^8^.

In a more recent study (2014) conducted in the same city, APMT did not show a good association with the percentage of weight loss (p = 0.113), AMC (p = 0.806) and AMAc (p = 0.770), but correlated significantly with AC (p = 0.003), TS (p = 0.000) and BMI (p = 0.000). APMT was also associated with gender and age, as there was a high prevalence of malnutrition in women and the elderly. The values ​​found in the DH were higher, suggesting a more rapid atrophy of this musculature in case of inactivity resulting from malnutrition^9^.

Study conducted in Pelotas (RS) published in 2015 identified an association and a significant linear trend between APMT values ​​and GSA categories (p < 0.001). It also found a strong association between APMT results for muscle mass depletion and GSA for malnutrition (p < 0.001). In this study, APMT showed low sensitivity (DH 34.9% and NDH 37.7%), and high specificity (greater than 90%) for predicting malnutrition^10^. 

In the results of a research carried out in Vitória (ES) in 2016, APMT showed to be correlated with BMI, AMC, AMAc, CC (p < 0.01) and GSA (p = 0.026), with AMC (p = 0.036) as the variable that most influenced APMT values^11^. In a study carried out in the city of Salvador (BA) in the year of 2016, APMT did not present a significant association with the presence of complications in the PP in either hand (DH p = 0.217 and NDH p = 0.148). However, the APMT of the NDH was significantly associated with specific infectious complications (p = 0.030)^12^. In another study conducted in the same year in Recife (PE), APMT was compared to the gold-standard GSA method, but did not present a significant association (DH p = 0.513 and NDH p = 0.842)^13^ ^).^

Data regarding the objectives, number of patients and age group, objective and subjective evaluations used for comparison with APMT and conclusion of the studies performed in surgical patients are described in the Figure 3. 

**Figure 2:**
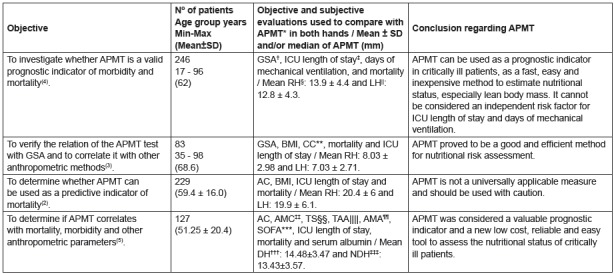
Characteristics of the studies performed in critical patients

Legend: *APMT - Adductor Pollicis Muscle Thickness; †GSA - Global Subjective Assessment; ‡ICU - Intensive Care Unit; §RH- Right Hand; ||Left Hand; ¶BMI- Body Mass Index; **CC - Calf Circumference; ††AC - Arm Circumference; ‡‡AMC - Arm Muscle Circumference; §§TS - Triceps Skinfold; ||||TAA - Total Arm Area; ¶¶AMA - Arm Muscle Area; ***SOFA - Sequential Organ Failure Assessment, †††DH - Dominant Hand; ‡‡‡NDH - Non-Dominant Hand.

**Figure 3:**
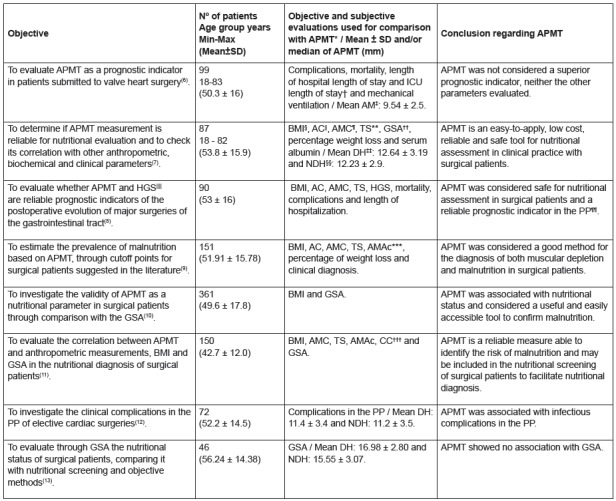
Characteristics of studies performed in surgical patients

Legend: *APMT - Adductor Pollicis Muscle Thickness; †ICU - Intensive Care Unit; ‡BH - Both Hands; §BMI- Body Mass Index; ||AC - Arm Circumference; ¶AMC - Arm Muscle Circumference; **TS - Triceps Skinfold; ††GSA - Global Subjective Assessment; ‡‡DH- Dominant Hand; §§ Non-dominant hand; ||||HGS - Hand Grip Strength; ¶¶PP - Postoperative Period; ***AMAc - Arm Muscle Area corrected; †††CC - Calf Circumference.

Other studies were performed with oncological and nephropathic patients, being the first of them performed in 2012 in the city of Porto Alegre (RS), with cancer patients. The APMT values ​​found in this study were associated with mortality (p < 0.001), however there was no association with length of hospital stay (DH p = 0.42 and NDH p = 0.43)^14^. Two studies carried out in Belo Horizonte (MG) in the year 2013 found the same result for APMT, which presented a significant difference between nourished and malnourished patients (p < 0.05), but a low agreement with the GSA (ƙ < 0.20); only one study had no association with the Glasgow prognostic score (p > 0.05)^15^ ^,^ ^16^.

The next group includes nephropathic patients with Chronic Kidney Disease (CKD) undergoing dialysis and is described below. The study carried out in Fortaleza (CE) in the year 2012 followed patients for 12 months and found a significant difference and positive correlation between APMT and BMI (r = 0.37; p 0.0001), AC (r = 0.437; p < 0.0001), AMC (r = 0.494; p < 0.0001), AMA (r = 0.449; p < 0.0001), percentage of weight adequacy (r = 0.355; p = 0.000), creatinine (r = 0.230; p = 0.006), albumin (r = 0.207; p = 0.013), percentage of body cell mass (r = 0.293; p=0.000) and FA (r = 0.402; p < 0.0001), and negative correlation with resistance measured by EBI (r = 20.403; p < 0.0001). However, no significant association was found with age, dialysis time, TS, hemoglobin, and reactance. Higher risks of hospitalization within six months and mortality were associated with lower APMT values^17^.

**Figure 4:**
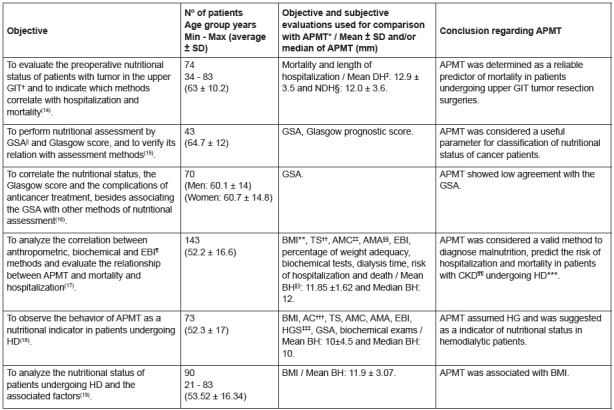
Characteristics of the studies performed in oncological and nephropathic patients.

Legend: *APMT - Adductor Pollicis Muscle Thickness ; †GIT - Gastrointestinal Tract; ‡DH - Dominant Hand; §MND - Non-Dominant Hand; ||GSA- Global Subjective Assessment; ¶EBI - Electrical Bioimpedance; **BMI - Body Mass Index; ††TS - Triceps Skinfold; ‡‡AMC - Arm Muscle Circumference; §§AMA - Arm Muscle Area; ||||BH- Both Hands; ¶¶CKD - Chronic Kidney Disease; ***HD - Hemodialysis; †††AC - Arm Circumference; ‡‡‡HGS - Hand Grip Strength.

A study carried out in São Paulo (SP) in the year of 2013 found APMT to be positively correlated with HGS (p < 0.05), serum albumin (p = 0.07), percentage of body cell mass (p < 0.05), reactance (p < 0.05) and FA (p < 0.05). However, APMT was not correlated with serum creatinine (p = 0.08), GSA (p = 0.55) and the anthropometric measures BMI (p = 0.64), AC (p = 0.62), AMC (p = 0.70) and AMA (p = 0.89)^18^. In 2014, in a study performed in a reference hospital in Rio Grande do Sul, APMT was associated with BMI (p = 0.001), where higher values ​​were more prevalent in overweight individuals and the lowest values ​​in those with low weight^19^. The objectives, number of patients and age group, objective and subjective evaluations used for comparison with APMT and completion of the work with oncological and nephropathic patients are described in the Figure 4.

The last group of studies described was performed in nephropathic, mostly ambulatory, patients and conducted in the city of Porto Alegre. The first study was published in 2012 with ambulatory cirrhosis patients and found a low percentage of malnutrition (14.3%) according to APMT measurement when compared to the HGS, TS, AC and AMC methods. APMT was not associated with disease severity and showed poor agreement with HGS methods [Kappa index (ƙ) = 0.12] and ASG (ƙ = 0.25)^20^. Another study found APMT to be significantly associated only with disease severity (p < 0.05), but also showed a low prevalence of malnutrition, a weak association with GSA (ƙ = 0.222) and no association with the diagnosis of malnutrition and inadequate dietary intake^21^. Two studies conducted in 2015 and 2016 with non-cirrhotic hepatitis C patients and patients before and after hepatic transplantation, respectively, did not identify any individuals with malnutrition and, therefore, APMT presented poor performance^22^ ^,^ ^23^.

In the year 2016, a study conducted in the city of Botucatu (SP) in cirrhotic patients with Hepatic Encephalopathy (HE) related APMT to disease severity, so that the reduction of 1 mm in the measurement was associated with an increase of 30.7% in the degree of HE (p = 0.0177). The lower values ​​were related to lower states of mental acuity, since measures lower than the cutoff point (6.5mm) were associated with the degrees I and II of HE (p = 0.013)^24^. 

**Figure 5:**
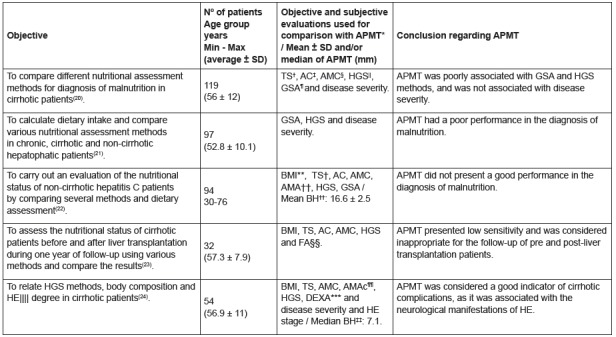
Characteristics of the studies performed in hepatopathic patients

Legend: *APMT - Adductor Pollicis Muscle Thickness; † TS - Triceps Skinfold; ‡ AC - Arm Circumference; § AMC - Arm Muscle Circumference; || HGS - Hand Grip Strength; ¶ GSA - Global Subjective Assessment; **BMI - Body Mass Index; ††AMA - Arm Muscle Area; ‡‡BH - Both Hands; ||||HE - Hepatic Encephalopathy; ¶¶AMAc - Arm Muscle Area corrected; ***DEXA - Dual Energy X-ray Absorptiometry.

Data regarding the objectives, number of patients and age group, objective and subjective evaluations used for comparison with APMT, as well as the conclusion of the studies performed in hepatopathic patients are described in the Figure 5.

## Discussion

APMT showed to be a good anthropometric parameter in critically ill patients without edema^3^ ^-^ ^5^, which may be justified by the greater expression of critical and acute conditions in the ICU, protein depletion and muscle loss caused by both the decrease in daily activities and the hypercatabolic status of these patients^25^. APMT was also considered a good prognostic indicator for mortality in this group^4^ ^,^ ^5^, being efficient even in patients with hand edema in one of the studies^4^, although it was not considered as a good prognosis for ICU length of stay^2^ ^-^ ^4^. Perhaps, this may be explained by the fact that APMT evaluates the lean mass and whose preservation is an indication of good evolution in such patients, since malnutrition contributes to increased mortality^25^. No justification was reported as to the usefulness as a good indicator for the ICU length of stay, but we believe that other clinical conditions are more determinant for the hospital stay. 

The limitations found in the studies included in this review were the interdiction of the adductor pollicis muscle area, and especially the presence of edema that diminishes the accuracy of the APMT measurement and, therefore, led to the exclusion of many patients, compromising the application of this measure in the practice. Thus, the researchers suggest that this measure must becarefully evaluated and applied before the onset of edema, ideally on the first day of ICU admission and analyzed in conjunction with the other anthropometric and GSA methods^2^ ^,^ ^5^. The combination of several tools may be more efficient to detect abnormality in the body composition of these patients^3^.

In the group of individuals submitted to surgeries, APMT also proved to be a good method for nutritional evaluation and diagnosis of malnutrition, but it was not considered a good prognostic factor for the prevalence of complications in the PP. Malnutrition in these patients is associated with surgical stress, as this procedure promotes the release of catabolic hormones that cause the degradation of muscle proteins, which is aggravated according to the nutritional deficit of the patient^9^. Thus, the potential detection of malnutrition can be justified by the good specificity of APMT verified in the studies, a characteristic that indicates a low rate of false positive results^7^ ^,^ ^10^. Furthermore, there is a correlation of APMT with anthropometric measures that happens due to the similar nature of the measures^7^. As for the non-association with PP complications, it is assumed that this result comes from the variability of this condition in function of the underlying disease severity and nutritional status prior to surgery.

In the evaluation of cancer patients, APMT was considered a good parameter for classification of nutritional status, but had low association with GSA^15^ ^,^ ^16^. This finding was indicated by the fact that GSA detected early functional alterations, while APMT detected malnutrition and changes in body composition only in later instance^15^ ^,^ ^16^. Only one study evaluated APMT as a prognostic indicator and found it to be a good parameter of mortality, resulting from its high discriminative power^14^.

With regard to nephropathic patients, APMT was also considered a good method of nutritional assessment compared to anthropometric parameters^17^ ^,^ ^19^ and a good predictor of hospitalization and mortality in the only study that evaluated its prognostic indication^17^, but the number of studies with this group is still small^17^ ^,^ ^18^. This outcome is based on the peculiarities of CKD patients, namely, reduced food intake and metabolic acidosis combined with uremia, factors that lead to protein catabolism^19^, which along with physical inactivity, further aggravates the disease^17^. Furthermore, malnutrition is per se a risk factor for death in hemodialysis patients^17^. 

 However, studies evaluating hepatopathic patients revealed poor performance of APMT in the diagnosis of malnutrition, and only one showed an association with neurological complications in HE. Two studies differed in relation to the severity of the disease. Non-association of APMT with malnutrition can be explained by fluid retention^24^, low sensitivity demonstrated by the method^23^ and anatomical muscle alteration that follows functional changes^21^.

An association of APMT with HE complications and disease severity was observed, most likely explained by the marked depletion of arm muscle experienced by cirrhotic patients^24^. The study in which there was no association with disease severity was justified by the low number of patients in the severe stage^20^.

GSA is present in almost half of the studies comparing different measures with APMT results. GSA is set as the gold standard by the American Society of Parenteral and Enteral Nutrition (ASPEN) for evaluation of hospitalized patients, and it is frequently used for validation of new methods^8^ ^,^ ^25^. GSA evaluates the nutritional status as a whole, including anthropometric, clinical, physical, metabolic and dietary data, identifying suspected or established malnutrition before changes in body composition. Thus, GSA presents the best prediction for arising complications; however, its efficacy depends on the ability of the evaluator to detect significant nutritional changes. This have led to the need to study new, simpler and less invasive methods to speed up the nutritional screening process^10^ ^,^ ^11^ ^,^ ^15^ ^,^ ^25^.

Among the ten studies comparing GSA with APMT, seven found a positive association between them and only three did not find association. Among the groups where association was detected are the surgical^7^ ^,^ ^10^ ^,^ ^11^, critical^3^ ^,^ ^4^ and oncological^15^ ^,^ ^16^ patients, and those where association was absent were studies with surgical^13^, nephropathic^18^ and hepatopathic^20^ patients.

Studies with healthy individuals were excluded from this review because they aimed to establish reference values ​​for this population^26^ ^,^ ^27^, and the research with institutionalized elderly people analyzed their nutritional profile, but not its effectiveness compared to other methods. It is important to emphasize that in the latter group, all patients presented values ​​within the normal range for healthy people and more studies are therefore necessary for the purpose of comparison^28^.

The greatest limitation observed in this review regarding the evaluation of APMT measurement was the absence of reference standards for the different clinical conditions, according to gender and age group. The values ​​often referred to in the studies come from healthy populations or are defined based on a single cutoff point, and consequently may underestimate malnutrition in adults and in men, and overestimate it in the elderly and women^8^. Another limitation found in the researched literature was the use of different adipometers and the standardization of the technique itself, which may have led to very different APMT values, besides the variability stemming from different evaluators^2^ ^,^ ^11^.

Minimum and maximum mean APMT values (mm) were, respectively, 7.03 ± 2.71 (LH) and 20.4 ± 6 (RH) in critically ill patients^2^ ^-^ ^5^; 12.0 ± 3.6 (NDH) and 12.9 ± 3.5 (DH) in cancer patients^14^; 10 ± 4.5 (BH) and 11.9 ± 3.07 (BH) in nephropathic patients^17^ ^-^ ^19^; 9.54 ± 2.5 (BH) and 16.98 ± 2.80 (RH) in surgical patients^6^ ^,^ ^7^ ^,^ ^12^ ^,^ ^13^ ^);^ and 16.6±3.5 and a median of 7.1 in hepatopathic patients^22^ ^,^ ^24^. However, these data refer to only 14 studies that provided such values. Yet, it is interesting to note that the lowest APMT values ​​were found in a study with critical patients (7.03 ± 2.71 in the left hand)^3^ and in hepatopathic patients (median of 7.1 mm in both hands)^24^.

Thus, it is necessary to determine specific cutoff points for pathologies or groups of individuals and carefully interpret the results. It is also necessary to assess the actual sensitivity and specificity of APMT. In this sense, sensitivity is a parameter that evaluates the probability of the result to be positive, and specificity analyzes the probability of that same result to be negative^29^.

## Conclusion

APMT was considered a good anthropometric parameter in the great majority of clinical conditions evaluated, except in patients with liver disease, and presented low sensitivity and high specificity. It was also indicated as a good prognostic indicator for mortality in critical, oncological and renal patients, and as a predictor of neurological complications in hepatic encephalopathy. However, further investigation of its prognostic usefulness in other clinical conditions, standardization of cutoff points for reference in the measurement classification and assessment of sensitivity and specificity is required. 
